# Supplementary material for: Bayesian insights into exchange and restriction in gray matter diffusion MRI
Source: Imaging Neurosci (Camb). 2026 Jul 27;4:IMAG.a.1317. doi: 10.1162/IMAG.a.1317 (PMC13409282; doi:10.1162/IMAG.a.1317)
Supplement: Supplementary Material [file IMAG.a.1317_supp.pdf]

Supplementary Material

NLLS Fitting Performance

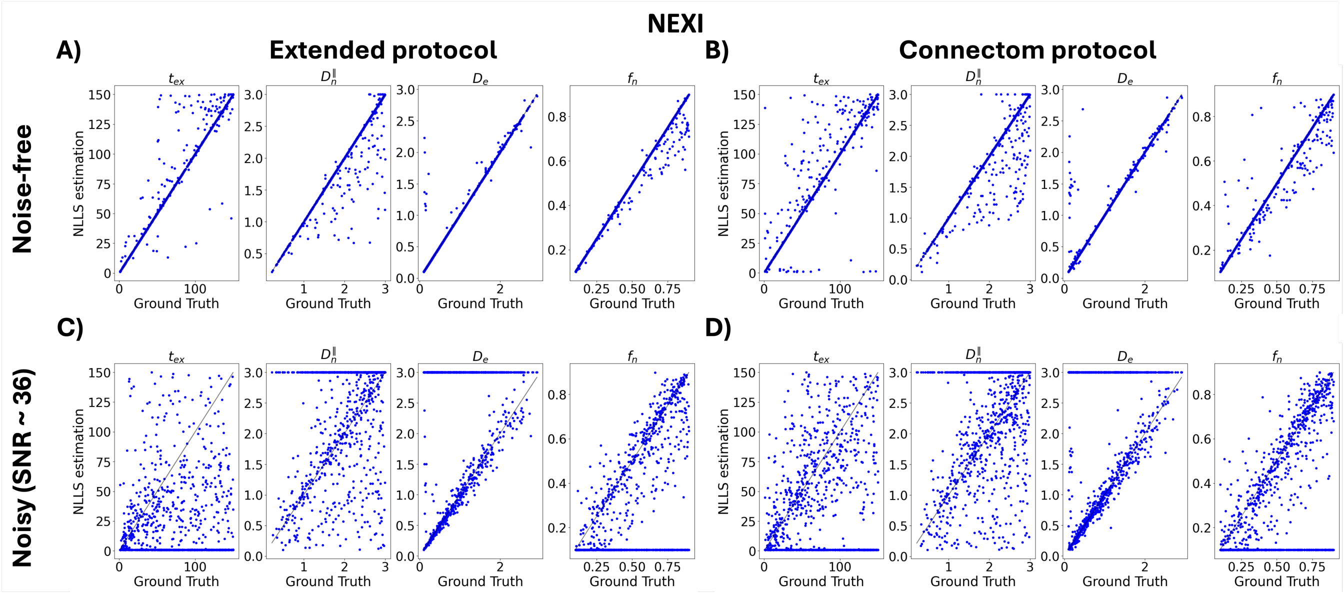

Figure 1: Fitting results for the NEXI model using NLLS on 1000 test simulations. Results are shown for the extensive *ex vivo* acquisition protocol (A & C), and the NEXI 3T Connectom protocol (B & D). In each subplot, we plot the estimates of the model parameters against their ground truth values.

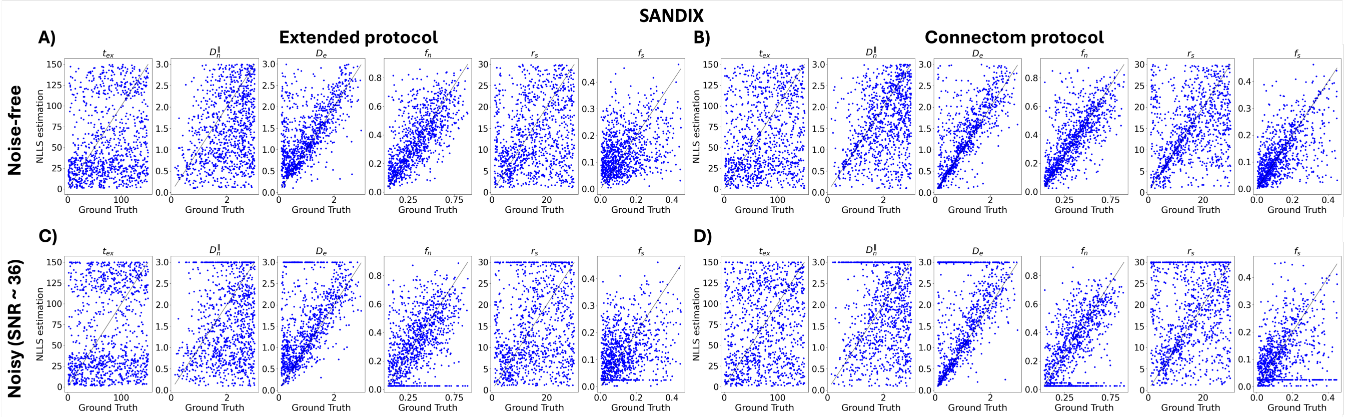

Figure 2: Fitting results for the SANDIX model using NLLS on 1000 test simulations. Results are shown for the extensive *ex vivo* acquisition protocol (A & C), and the NEXI 3T Connectom protocol (B & D). In each subplot, we plot the estimates of the model parameters against their ground truth values.

Time Comparison between μGUIDE and NLLS

Table 1: Time for fitting 1000 simulations using μGUIDE or NLLS, on both protocols, with and without noise. Time reported excludes the training time for μGUIDE and the initialization for NLLS. Fittings were performed on CPU for both methods (32 cores).

		Extensive *ex vivo* acquisition protocol		NEXI 3T Connectom protocol	
		μGUIDE	NLLS	μGUIDE	NLLS
NEXI	Noise-free	5s	45s	9s	25s
	Noisy (SNR $\sim$ 50)	9s	1min 08s	9s	36s
SANDIX	Noise-free	9s	9min 15s	7s	3min 28s
	Noisy (SNR $\sim$ 50)	7s	10min 29s	6s	3min 22s

Scan-rescan Comparison between μGUIDE and NLLS

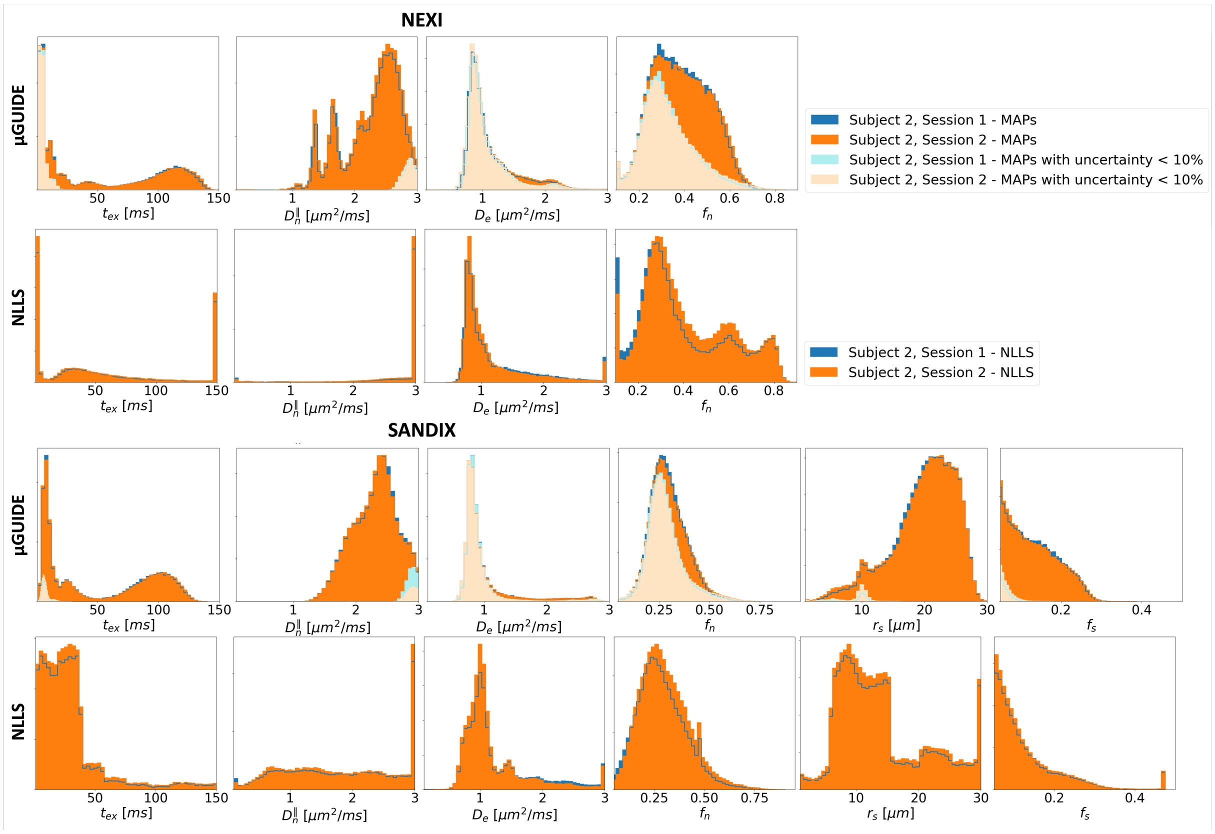

Figure 3: Comparison of parameter estimates across cortical ribbon from one participant on two sessions using μGUIDE and a NLLS method. μGUIDE estimates are thresholded based on posterior distribution uncertainty, with distributions shown for voxels with uncertainty below 10%.

Validation of Results Under Gaussian Noise

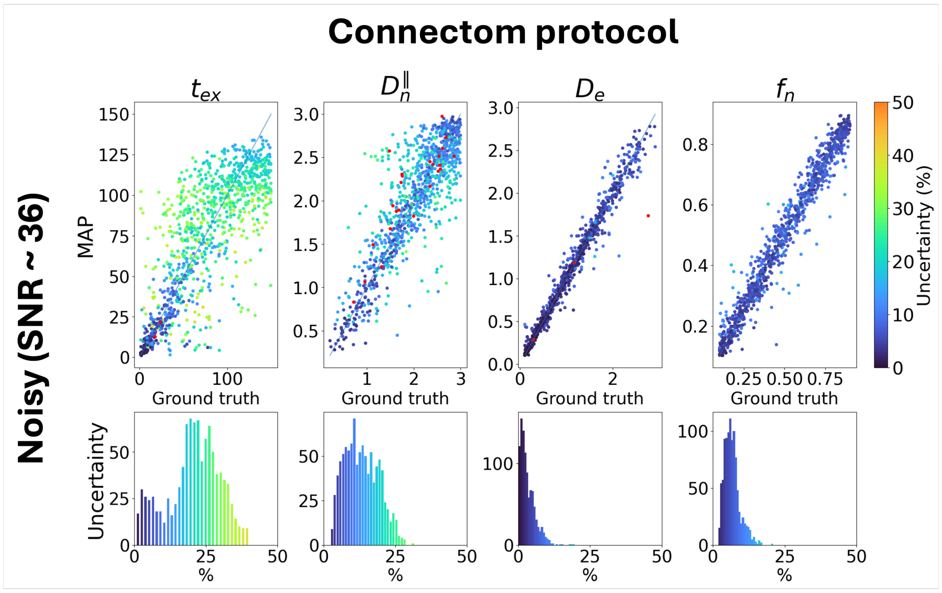

Figure 4: Fitting results for the NEXI model using μGUIDE on 1000 simulated signals generated according to the NEXI 3T Connectom protocol and corrupted with Gaussian noise. The top row displays the MAP estimates of the model parameters plotted against their ground truth values, color-coded by their uncertainty values. Red dots indicate cases where the posterior distribution was identified as degenerate. The bottom row shows the distribution of uncertainty values across all test simulations.
